# Exercise Intervention for REM Sleep Behavior Disorder in Parkinson's Disease: Mechanisms and Implications for Neurorehabilitation

**DOI:** 10.1002/cns.70991

**Published:** 2026-06-15

**Authors:** Xinhui Qiu, Zihui Wang, Ruixue Xu, Yiying Hu, Weidong Le

**Affiliations:** ^1^ Key Laboratory of Liaoning Province for Research on the Pathogenic Mechanisms of Neurological Diseases The First Affiliated Hospital of Dalian Medical University Dalian China; ^2^ Department of Neurology, Sir Run Run Shaw Hospital, School of Medicine Zhejiang University Hangzhou China; ^3^ Department of Neurosurgery The First Affiliated Hospital of Dalian Medical University Dalian China; ^4^ Department of Pharmacology and Chemical Biology Emory University School of Medicine Atlanta Georgia USA

**Keywords:** α‐synuclein, exercise therapy, Parkinson's disease, REM sleep behavior disorder

## Abstract

**Backgrounds:**

REM sleep behavior disorder (RBD) is a prodromal non‐motor symptom of Parkinson's disease (PD) and other α‐synucleinopathies, affecting approximately 30%–60% of PD patients. It is closely associated with α‐synuclein (α‐syn) aggregation, with its primary pathophysiology involving the impairment of brainstem circuits, resulting in movement disorders and circadian rhythm dysfunction. Although exercise is recognized for improving motor symptoms and delaying neurodegenerative disease progression, a comprehensive framework of its underlying mechanisms for RBD symptoms has not yet been thoroughly analyzed.

**Methods:**

This review examines current literature and evidence regarding the various mechanisms and neuroprotective effects of exercise interventions specifically targeting RBD and its associated symptoms.

**Results:**

Exercise exerts neuroprotective effects for RBD through multiple underlying mechanisms. The analysis highlights key pathways, including the enhancement of α‐syn clearance, the regulation of neurocircuits and circadian rhythms, the modulation of oxidative stress and mitochondrial function, and the reduction of neuroinflammation.

**Conclusion:**

Exercise demonstrates significant potential as a multi‐targeted intervention. By addressing these underlying pathophysiological mechanisms, exercise provides supportive benefits that may positively influence the disease trajectory for patients with RBD and PD.

## Graphical abstract

This review systematically explores exercise as a multi‐target intervention for RBD, highlighting its mechanisms of action and regulatory effects on key pathophysiological processes.

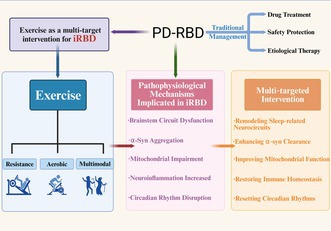

## 1 Introduction

**FIGURE 1:**
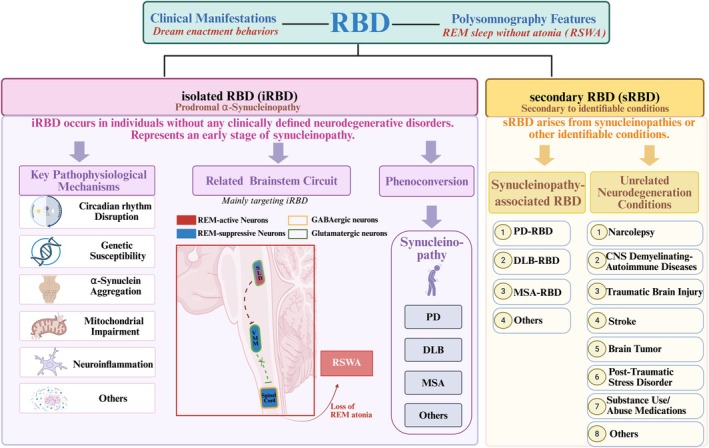
Clinical and pathophysiological framework of REM sleep behavior disorder (RBD). RBD is characterized by dream enactment behaviors and REM sleep without atonia (RSWA), the latter representing the core polysomnographic feature of the disorder. RBD can be broadly classified into isolated RBD (iRBD) and secondary RBD (sRBD). iRBD occurs in individuals without clinically defined neurodegenerative disorders and is considered an early prodromal stage of α‐synucleinopathy. The proposed pathophysiological mechanisms underlying iRBD include circadian disruption, genetic susceptibility, α‐synuclein aggregation, mitochondrial impairment, neuroinflammation, and dysfunction of REM sleep‐related brainstem circuitry involving GABAergic and glutamatergic pathways, ultimately contributing to the loss of REM sleep atonia and the development of RSWA. During disease progression, iRBD may undergo phenoconversion into α‐synucleinopathies such as Parkinson's disease (PD), dementia with Lewy bodies (DLB), and multiple system atrophy (MSA), in which RBD subsequently manifests as synucleinopathy‐associated secondary RBD. In addition, sRBD may also occur in association with other identifiable neurological, medical, or medication‐related conditions, including narcolepsy, CNS demyelinating‐autoimmune diseases, traumatic brain injury, stroke, brain tumors, post‐traumatic stress disorder, and substance use or medication exposure. DLB, dementia with Lewy bodies; GABA, gamma‐aminobutyric acid; iRBD, isolated REM sleep behavior disorder; MSA, multiple system atrophy; PD, Parkinson's disease; RBD, REM sleep behavior disorder; RSWA, REM sleep without atonia; sRBD, secondary REM sleep behavior disorder.

PD is a progressive neurodegenerative disorder with dopaminergic neuron loss in the substantia nigra and other brain regions. The pathological hallmark is the aggregation of Lewy bodies, and misfolded α‐syn is the major component [1]. The clinical features of PD include a classical motor syndrome (bradykinesia, rest tremor, rigidity, and postural instability) and various non‐motor symptoms [2]. Non‐motor symptoms are significant as prodromal markers in PD; they often precede the onset of motor features and significantly contribute to the disease burden and progression [3]. Rapid eye movement (REM) sleep behavior disorder (RBD) is characterized by dream enactment that occurs during REM sleep without atonia (RSWA). RBD is generally classified as isolated RBD (iRBD) and secondary RBD (Figure 1) [4]. iRBD refers to cases in the absence of neurological features but with a high risk (33% in 5 years, 90% in 14 years) of developing α‐synucleinopathy, which includes PD, dementia with Lewy bodies (DLB), and multiple system atrophy [5]. Secondary RBD may result from an acute or subacute‐onset central nervous system disorder based on focal brainstem lesions; the most common cause in the literature is neurodegenerative illness; others include vascular, demyelinating, autoimmune disorders and so on [6].

iRBD is the most specific prodromal marker of PD and other α‐synucleinopathies [7]. It is considered to be an early clinical sign and present in approximately 30%–60% of PD patients, which is associated with poor sleep quality, increased risk of injury to oneself or bed partners, and accelerated decline in cognitive and motor function [8]. PD patients who present with clinical symptoms of RBD are typically referred to as PD‐RBD [9]. The diagnosis of RBD is based on clinical features and polysomnography (PSG) [10]. RSWA is a key hallmark of iRBD in PSG, which refers to the loss of physiological muscle atonia during REM [11]. Evidence of PSG‐confirmed iRBD is the gold standard, with RSWA and dream enactment behavior [12]. The underlying pathology is associated with neuronal loss and structural alterations in the brainstem circuits responsible for REM sleep atonia [13]. However, the precise causes of neuronal dysfunction in these brainstem regions, along with the mechanisms driving the prodromal stage, remain incompletely understood. Recent research has focused on several potential contributing pathways, including circadian gene dysfunction, oxidative stress, mitochondrial impairment, neuroinflammation, autophagy‐lysosome dysfunction, and α‐syn prion‐like transmission [13].

The present studies have demonstrated the importance of reducing the risk of iRBD progressing to neurological disorders. Unfortunately, no treatment has been shown to affect the disease course in RBD. Clonazepam and melatonin are the most commonly used drugs to treat RBD [4]. They both reduce the frequency of unpleasant dreams and dream enactment behaviour [14]. However, clonazepam is limited by side effects such as dizziness and an increased risk of falls. Melatonin (6–18 mg at bedtime) can improve RBD symptoms in 70% of patients, but some placebo‐controlled trials using a lower dose showed no benefit [15]. Non‐pharmacological interventions provide valuable opportunities to slow down the pathological progression in RBD [16]. Physical exercise has been suggested as an effective lifestyle intervention for patients with neurodegenerative diseases [17], which may ameliorate the motor symptoms, cognitive impairment, psychiatric disorders, and sleep disorders [18].

In this review, we summarize recent advances in exercise interventions that may be able to alleviate RBD symptoms and delay neurodegenerative pathological progression. We aim to provide a comprehensive overview of the molecular, cellular, and neurophysiological mechanisms underlying RBD, and highlight the potential of exercise intervention strategies in PD‐RBD.

This review conducts a systematic search of articles published in English in the PubMed/Web of Science databases up to March 2026. The search strategy used combinations of the following keywords: “REM sleep behavior disorder”, “RBD”, “isolated REM sleep behavior disorder”, “iRBD”, “Parkinson's disease”, “synucleinopathy”, “exercise”, “physical activity”, “aerobic exercise”, “resistance training”, “tai chi”, “yoga”, “sleep”, “REM sleep without atonia”, “RSWA”, “alpha‐synuclein”, “mitochondrial impairment”, “neuroinflammation”, and “circadian rhythm”. In addition, the reference lists of relevant reviews and original articles were manually screened to identify additional eligible studies.

## 2 The Underlying Pathogenesis of RBD: RSWA Mechanisms and the Neurodegeneration

RSWA is defined as abnormal skeletal muscle activity during REM sleep, a stage in which muscle tone is normally reduced. RSWA is detected on PSG, characterized by persistent muscle tone during REM sleep. It can be shown as either sustained (tonic) excessive activity or intermittent (phasic) excessive activity in the chin or limb electromyography [19]. The RSWA reflects impaired brainstem neural networks and constitutes a key diagnostic criterion for RBD [20]. Pathological alterations within the brainstem circuits underlie the generation of RBD symptoms. As iRBD is an important prodromal feature of PD, it often shares pathophysiological processes with PD. Accumulating evidence suggests that neurodegeneration in focal brainstem lesions also contributes to the pathogenesis of iRBD, involving α‐syn‐related pathology, circadian dysregulation, mitochondrial impairment, neuroinflammation, and other neurodegenerative processes [21].

### 2.1 Neural Mechanisms Underlying RSWA

The pontomedullary pathway is essential for the generation of normal REM atonia. In rodents, neurons in the sublaterodorsal tegmental nucleus (SLD), considered homologous to the subcoeruleus region in cats and humans, play a central role in initiating REM atonia [22]. These predominantly glutamatergic neurons project to inhibitory neurons in the ventromedial medulla, including the gigantocellular reticular nucleus [23]. These medullary neurons conversely project to the second motor neurons in the brainstem and anterior horn of the spinal cord, resulting in skeletal muscle paralysis during REM sleep [24]. Lesions or functional disruption of this pontomedullary pathway produce RSWA and complex motor behaviors that resemble RBD in animal models [25]. Furthermore, human neuroimaging studies support the involvement of brainstem nuclei implicated in REM atonia regulation in patients with RBD, particularly in the context of synucleinopathies such as PD and DLB [19].

Multiple neurotransmitter systems, such as glutamatergic, gamma‐aminobutyric acid (GABA)‐ergic, and cholinergic pathways, participate in REM sleep regulation. The core REM atonia circuitry seems to operate in non‐dopaminergic modes [26, 27]. RBD symptoms are not usually improved by levodopa treatment, and current symptomatic treatment does not recommend levodopa as a standard therapy [28]. Only a few case–control studies show that dopamine transporter (DAT) binding is reduced in the striatum of iRBD patients, even in the prodromal stages through single‐photon emission computed tomography (SPECT) and positron emission tomography (PET) [29]. Reduced DAT binding is associated with increased muscle activity during REM sleep. In addition, the role of dopamine deficiency in maintaining REM atonia is still controversial [30].

### 2.2 Circadian Alterations in iRBD and PD‐RBD

Clinical studies have reported circadian abnormalities in both iRBD and PD, including altered peripheral clock gene expression, delayed melatonin phase, reduced nocturnal melatonin secretion, and disrupted rest activity rhythms [31]. In patients with iRBD from a case report, several core circadian clock genes, including period circadian regulator 2 (*PER2*), brain and muscle arnt‐like 1 (*BMAL1*), and nuclear receptor subfamily 1 group D member 1 (*NR1D1*), lost rhythmic expression in peripheral blood mononuclear cells (PBMC). In addition, the 24‐h melatonin spectra show phase delay and peak dispersion, suggesting a mismatch between the central pineal output and peripheral oscillators [32]. Consistently, saliva‐based assessments in iRBD patients have demonstrated disrupted rest‐activity rhythms, delayed dim‐light melatonin onset, and reduced nocturnal melatonin levels, providing further evidence of a disturbed melatonin circadian gene axis [30, 33, 34].

Similar circadian alterations have also been observed in PD patients. A large case–control cohort study reported that *BMAL1*, circadian locomotor output cycles kaput (*CLOCK*), cryptochrome circadian regulator 1 (*CRY1*), *NR1D1*, and *PER2* were downregulated in PBMCs, while melatonin levels were reduced [35]. Overall, these data suggest that abnormal central and peripheral circadian rhythms may disrupt the stability of REM sleep structure. However, these circadian abnormalities should be interpreted cautiously. Current evidence suggests that they reflect broader involvement of the sleep–wake system in iRBD and PD‐RBD, rather than primary mechanisms underlying RSWA. Circadian dysfunction may instead represent an indicator of neurodegenerative burden [36]; thus, direct evidence supporting a causal role in RSWA remains lacking.

### 2.3 Genetic Susceptibility in iRBD and Synucleinopathy

Genetic studies further support the notion that iRBD lies within the prodromal spectrum of synucleinopathy [37]. A genome‐wide association study involving 2843 cases of iRBD patients has identified three risk loci near SNCA, glucosylceramidase beta (GBA), and transmembrane protein 175 (TMEM175) [38]. Notably, the RBD‐associated signals at the SNCA and SCARB2 loci are distinct from the major PD risk variants, suggesting that RBD may represent a genetically and biologically specific subtype within the α‐synucleinopathy spectrum [39]. GBA variants increase the risk of iRBD, and they also increase the rate of conversion to neurodegeneration [40, 41]. Overall, currently available genetic evidence suggests that iRBD shares important molecular pathways with PD and DLB, particularly those involving α‐synucleinopathy and lysosomal biology. However, these genetic factors are better interpreted as contributors to susceptibility and progression rather than as direct mechanisms generating RSWA.

### 2.4 The Misfolded α‐Syn Aggregation

Misfolded α‐syn aggregates are the principal protein component of Lewy pathology and are thought to play a central role in the neurodegenerative processes underlying iRBD and related α‐synucleinopathies [42]. Post‐mortem studies indicate that individuals with iRBD exhibit α‐syn deposition and neuronal loss in brainstem structures involved in REM sleep regulation [43]. Furthermore, according to Braak's staging theory, the pathological process of α‐syn in PD may originate in the peripheral nervous system and the lower brainstem, then subsequently ascend to other brain regions [44]. The pontomedullary nucleus in the lower brainstem regulates REM sleep, leading to the loss of muscle atonia during this stage [45]. This “bottom‐up” pathological spread may occur before the onset of motor symptoms in PD [46]. This evidence strongly supports the role of early α‐syn pathology in the pathophysiology of iRBD and its progression toward dominant α‐synucleinopathies. In a longitudinal observational study of patients with iRBD, detection of misfolded α‐syn in CSF by real‐time quaking‐induced conversion (RT‐QuIC) was reported to predict phenoconversion to PD or DLB with a sensitivity of 95% and specificity of 100% over a 5‐year follow‐up period [47]. A larger cohort study further demonstrated elevated α‐syn seeding activity in the CSF of iRBD patients compared with healthy controls. Collectively, these clinical findings suggest an association between α‐syn pathology and disease progression in iRBD; however, further mechanistic studies are required to establish its causality and assess its clinical applicability [48].

### 2.5 Mitochondrial Impairment in RBD

Mitochondrial impairment has increasingly been implicated in the neurodegenerative process of iRBD and related synucleinopathies [49]. Mechanistically, mitochondrial impairment may reduce ATP production, increase reactive oxygen species generation, and enhance neuronal susceptibility to α‐syn‐related toxicity [45]. In postmortem PD patients, the mitochondrial respiratory chain was altered, and upregulated mitochondrial subunits were found in GABAergic and glycinergic neurons in the pedunculopontine nucleus, a crucial area implicated in RBD [50] and network fragmentation [51]. A cohort study reported increased levels of deletion‐bearing mitochondrial DNA (mtDNA) in the CSF and serum of patients with iRBD compared with healthy controls [49]. Another study reported increased glycolysis activity and reduced mitochondrial respiration in PBMCs from iRBD patients, with a diagnostic accuracy of 82% for predicting conversion to PD [52]. Taken together, these findings support mitochondrial impairment and oxidative stress as important contributors to the upstream pathological process of iRBD. These may increase neuronal vulnerability and contribute to degeneration within brainstem and extra‐brainstem networks.

### 2.6 Neuroinflammation in RBD

Neuroinflammation is another early‐stage key feature in iRBD. PET imaging demonstrated a significant increase in microglia activation in the substantia nigra, which was associated with reduced dopaminergic function in iRBD patients [53]. In addition to central inflammatory changes, peripheral immune alterations have also been reported in iRBD. Increased expression of CD116 in blood monocytes and reduced expression of the human leukocyte antigen–DR isotype suggest dysregulated peripheral immune responses [54]. Besides, the level of the inflammatory cytokine, interleukin‐10, in the plasma of iRBD patients was significantly upregulated [55]. In the mouse model, α‐syn can induce neuroinflammation and neuronal death in the brain [56]. Overall, these findings support the presence of both central and peripheral immune dysregulation in iRBD. Quantitative neuroimaging studies have provided in vivo evidence of neuroinflammation in this condition. In a case–control PET study using [^11^C] PK11195 (a marker of microglial activation) in iRBD patients, a significant increase in tracer binding in the substantia nigra [53]. However, these studies are limited due to a relatively small sample size; larger prospective cohorts are needed to validate these findings.

Above all, α‐syn aggregation, mitochondrial impairment, and neuroinflammation are increasingly recognized as interconnected processes. Aggregated α‐syn has been shown to impair mitochondrial complex I activity and disrupt mitophagy, leading to increased reactive oxygen species production, which in turn promotes α‐syn misfolding and propagation [57]. Conversely, α‐syn can activate microglial NOD‐like receptor family pyrin domain containing 3 (NLRP3) inflammasomes, contributing to a pro‐inflammatory environment that exacerbates mitochondrial damage [58, 59]. This pathogenic triad is detrimental to the brainstem nuclei, which are also highly susceptible to α‐syn pathology and oxidative stress [43, 59]. Moreover, the post‐transcriptional regulation by microRNAs (miRNAs) may further modulate these pathways in PD. For example, miRNAs such as miR‐7 and miR‐153 have been shown to negatively regulate α‐syn expression, whereas others (e.g., miR‐155, miR‐146a) are reported to modulate the neuroinflammatory responses and mitochondrial function in synucleinopathies [60].

## 3 Management Strategies of RBD

The current management of RBD is primarily symptomatic and focuses on reducing dream enactment behaviors and minimizing the risk of sleep‐related injury [4]. The first‐line drug treatment is clonazepam, which is a long‐acting benzodiazepine [61]. Its mechanism involves enhancing GABAergic inhibition and reducing the output of spinal motor neurons [28]. The effect of clonazepam in RBD patients has been shown to reduce motor symptoms during REM sleep, but it cannot decrease dream content [62]. Thus, clonazepam provides symptomatic relief but does not alter disease progression [15]. Meanwhile, melatonin typically exhibits good tolerance in improving REM sleep atonia [63]. Its mechanism involves regulating circadian rhythms, enhancing inhibition of motor activity, and stabilizing REM sleep structure [64]. Case reports are effective in RBD treatment. However, further placebo‐controlled clinical studies are needed [65]. In addition, there is currently insufficient data to support the use of anticholinergics, dopamine agonists, and antipsychotics for RBD patients [66]. Other drugs, such as selective serotonin reuptake inhibitors (SSRIs), diazepam, and lorazepam are controversial [67]. For the treatment of deep‐brain stimulation for PD combined with RBD patients, the outcome was limited. Some cases showed it may increase RBD symptoms after surgery in subthalamic‐stimulated patients and decrease in pallidal‐stimulated patients [68].

In addition to those interventions, behavioral strategies are essential components of comprehensive RBD management. The American Academy of Sleep Medicine recommends taking safety measures to reduce the risk of injury during nocturnal episodes. It is crucial to remove sharp objects, fill bed areas, and place the mattress on the floor [15, 66]. Maintaining a regular sleep–wake schedule, avoiding alcohol consumption, reducing sleep deprivation, practicing emotional control, ensuring caregiver supervision, and making lifestyle modifications are all helpful in enhancing sleep quality and safety as well [69]. Notably, exercise has emerged as an effective non‐pharmacological intervention. Clinical and experimental studies suggest that exercise can enhance α‐syn clearance, reducing oxidative stress and neuroinflammation, regulating neural circuits and circadian rhythm to slow the progression to neurodegenerative diseases [70, 71].

## 4 Clinical and Biological Rationale for Exercise in RBD‐Related Disorders

Different forms of exercise, including aerobic exercise, resistance training, and multimodal exercise, may provide supportive benefits in RBD‐related conditions through complementary mechanisms.

### 4.1 Aerobic Exercise

Aerobic exercise interventions are widely recognized as a therapy method for PD patients to improve motor symptoms and sleep disorders. It is a continuous physical activity that involves the large muscles, increases heart rate and calorie demand, enhances cardiovascular health, and improves oxygen utilization efficiency [72]. Both high‐intensity (by 70%–90% of maximal heart rate) and moderate‐intensity aerobic exercise (by 50%–70% of maximal heart rate) can improve general sleep‐related outcomes and enhance neuroplasticity, although direct evidence of benefit in iRBD patients remains limited [70]. A systematic review involving participants found that, following an exercise intervention, sleep quality, nocturnal restlessness, and nocturnal dreams in PD patients showed significant improvements [73]. In addition, after 16 weeks of high‐intensity aerobic exercise, sleep efficiency and sleep structure dysfunction in PD were markedly improved, including increased total sleep time and decreased nocturnal awakenings. Furthermore, for 3–6 months, home‐based aerobic exercise in early PD has been shown to significantly reduce the progression of motor symptoms, increase neuroplasticity, and enhance dopamine release [74]. Based on this evidence, it is recommended to perform a practical aerobic exercise regimen for individuals with iRBD, including 30–45 min per session, at least 3 times per week, at an intensity above 70% of maximal heart rate if tolerated [75].

Evidence from animal studies also showed that aerobic exercise intervention modulates sleep architecture. After 6 weeks of voluntary exercise, the REM sleep time was elevated in stress‐induced rats by reducing disruptions in the circadian rhythm [76]. Additionally, running wheel exercise can diminish slow‐wave activity and regularize sleep structure in older mice [77]. The frequency of EEG theta power in REM sleep is altered after exercise, a phenomenon associated with hippocampal CA1 neuronal activation [78]. In human α‐syn transgenic mice, after 2 months of running exercise, the spread of α‐syn was reduced by activating the proliferator‐activated receptor α to stimulate lysosomal biogenesis [79]. In the MPTP‐induced PD mouse model, exercise performed over 2 months significantly reduced α‐syn levels in the brain through increased expression of silent information regulator transcript 1 (SIRT1), enhanced mitochondrial biogenesis, and increased respiratory chain enzyme activities [57]. In summary, aerobic exercise may improve sleep architecture, enhance neuroplasticity, activate molecular pathways such as SIRT1, and reduce α‐syn aggregation, supporting its potential role as a biologically relevant strategy for iRBD.

### 4.2 Resistance Training

Resistance training is designed to enhance muscular strength, size, and power by applying an external load (more than 70% of the one‐repetition maximum load) [80]. Unlike aerobic exercise, resistance training releases myokines [80], increases short‐term dopamine transport across the blood–brain barrier, and improves motor symptoms in PD [81]. Clinical research demonstrated that PD patients who underwent resistance training twice a week for 12 weeks significantly improved their sleep quality. The sleep latency, sleep duration, sleep disturbance, and daytime dysfunction were altered considerably, more closely resembling those of healthy controls [82]. In older individuals, long‐term resistance training consistently reduces systemic inflammation factors, which are associated with increased muscle mass [83]. This anti‐inflammatory effect may counteract the chronic neuroinflammation observed in early iRBD, potentially reducing microglial activation and exposure to inflammatory cytokines in brainstem regions susceptible to the pathological effects of α‐syn [84]. Furthermore, muscle contraction releases exosomes that contain lactic acid, proteins, and microRNAs via endocrine, paracrine, and autocrine pathways to enhance neurogenesis and synaptic plasticity [85]. In another objective study, after a 16‐week resistance training intervention program in PD patients, the PSG showed that sleep efficiency and slow‐wave sleep had significantly improved [75]. This enhanced slow‐wave and REM sleep is positively correlated with serum levels of brain‐derived neurotrophic factor (BDNF), which are higher after exercise [86]. Above all, resistance training may help preserve neuromuscular function and support broader health‐related outcomes, but large‐scale, high‐quality studies of exercise as an intervention in RBD, especially iRBD, are still needed.

### 4.3 Multimodal Exercise

Other multimodal exercise programs have been shown to improve physical function, quality of life, and reduce sedentary behavior [87]. In the early stages of PD, a comprehensive exercise plan that combines aerobic, resistance, and balance training has been shown to slow the progression of both motor and non‐motor functions effectively. In clinical trials, Argentine tango, Sardinian dance, yoga, and tai‐chi have demonstrated significant improvements in motor symptoms, gait, balance, and sleep quality [88, 89, 90, 91, 92]. It has been found that tai‐chi and yoga, as low‐intensity physical exercises, can activate the vagus nerve, regulate the balance between the sympathetic and parasympathetic nervous systems, and ameliorate autonomic function [93]. In the early stages of PD, RBD syndrome is associated with a decrease in sympathetic dominance states [94]. These low‐intensity exercises reduce the sympathetic nervous system and elevate nocturnal heart rate variability during REM sleep, which may support sleep‐related and autonomic regulation in patients with iRBD [95]. Other exercise programs, including group boxing training, agility training, pilates, and personalized home exercise plans, have shown potential benefits for PD patients [96, 97, 98]. However, further investigation is needed in the research design to determine the effectiveness of these interventions in iRBD patients [79]. Because little RCT research is enrolled in, iRBD patients with PSG‐/RSWA as a primary endpoint, existing clinical studies have several limitations: most have been conducted in patients with PD rather than iRBD; sample sizes are relatively small (29–130 participants); only a small number of patients are employed PSG; and RSWA is not quantitatively assessed as a primary outcome.

## 5 Potential Mechanisms of Exercise Ameliorate RBD Symptoms

**FIGURE 2:**
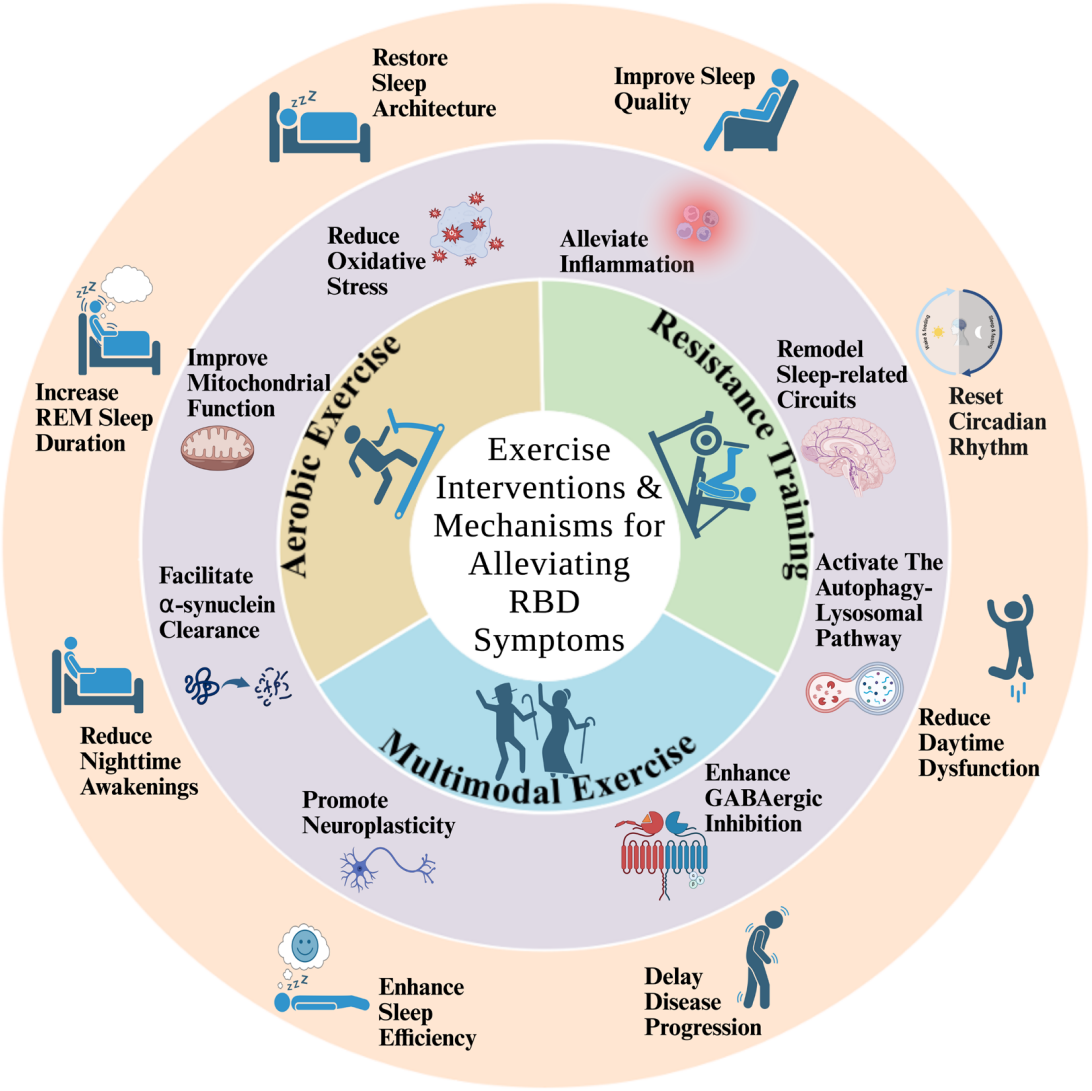
Potential mechanisms and therapeutic effects of exercise interventions in alleviating REM sleep behavior disorder. Current exercise interventions for REM sleep behavior disorder, including aerobic exercise, resistance training, and multimodal exercise, may exert beneficial effects through shared and multi‐target regulatory mechanisms. These interventions may improve mitochondrial function, facilitate α‐synuclein clearance, reduce oxidative stress, and alleviate neuroinflammation, promote neuroplasticity and GABAergic inhibitory function, activate the autophagy lysosomal pathway, and remodel sleep‐related neural circuits. Together, these biological effects may improve sleep quality and sleep architecture, increase sleep efficiency, reduce nocturnal awakenings and daytime functional impairment, prolong REM sleep duration, and potentially delay the progression of RBD and related neurodegenerative diseases.

The potential benefits of exercise in RBD‐related conditions may be mediated by molecular and cellular pathways, which are systematically categorized and summarized in Figure 2 and Table 1.

**TABLE 1:** List of the underlying mechanisms in exercise modalities RBD.

Exercise classification	Exercise forms	Model	Study design & sample size	Main mechanism	Pathway	Effects	Ref
Aerobic exercise	Walking, cycling, running	Chronic MPTP/probenecid‐induced mouse model of PD/A53T α‐synuclein PD mouse models	Animal study, 8–10 mice/group (MPTP/p); 5–8 mice/group (A53T)	Increase α‐Synuclein Clearance	Activates autophagy‐lysosomal pathway (AMPK/PINK1‐mediated); enhances UPS activity; reduces exosome‐mediated propagation	α‐syn clearance ↑; aggregate formation ↓; intercellular transmission ↓	[56, 76, 99, 100, 101, 102, 103, 104, 105]
Aerobic exercise	Walking, cycling, running	MPTP/probenecid‐induced mouse model of PD/6‐OHDA‐lesioned rats	Animal study, 8–10 mice/group (MPTP/p); 8 rats/group (6‐OHDA)	Oxidative Stress & Mitochondrial Function	Enhances mitochondrial biogenesis and mitophagy; activates SIRT1 pathway; improves mitochondrial dynamics	Antioxidant capacity ↑; ROS ↓; mitochondrial protection ↑; renewal and functional recovery ↑	[104, 105, 106, 107, 108, 109, 110]
Aerobic exercise	Brisk walking, cycling, running	Rotenone‐induced PD mouse model/PFF‐seeded mouse model	Animal study/Systematic review, 10 mice/group (rotenone); 8–10 mice/group (PFF); systematic review: 16 animal studies	Neuroinflammation Modulation	Suppresses NLRP3 inflammasome via TLR4/MyD88/NF‐κB pathway; modulates microglial activation; increases BDNF/TrkB signaling	Neuroinflammation ↓; neuronal protection ↑; BDNF signaling ↑	[57, 105, 106]
Aerobic exercise	Brisk walking, cycling, running	MPTP‐induced PD mouse model/6‐OHDA‐lesioned rat model	Animal study, 8–10 mice/group (MPTP); 8 rats/group (6‐OHDA)	Neurocircuit Remodeling	Enhances GABAergic activity in SLD; normalizes circadian gene expression; promotes BDNF/TrkB signaling; improves functional connectivity	Sleep architecture stabilization↑; neuroplasticity↑; relevance to REM‐atonia‐related circuitry	[22, 23, 24, 96, 97, 98, 111, 112]
Resistance training	Dumbbell training, weightlifting	PD clinical populations	RCT, *n* = 22 (11 resistance training, 11 control)	Sleep Architecture & Neuromuscular Improvement	Promotes release of myokines and exerkines; increases BDNF expression; modulates systemic inflammation	Sleep efficiency ↑; slow‐wave sleep ↑; daytime dysfunction ↓; inflammatory burden ↓	[79, 80, 81, 82, 83]
Resistance training	Dumbbell training, weightlifting	PD clinical populations	RCT, *n* = 40 (20 resistance training, 20 control)	Dopaminergic & Motor Enhancement	May enhance short‐term dopamine transport across BBB; improves muscle strength and motor control	Dopaminergic function ↑; motor symptoms ↓	[77, 80]
Multimodal exercise	Tango dance, yoga, tai chi, qigong, group boxing	PD clinical populations	Systematic review (multiple RCTs)/Cross‐sectional, cross‐sectional: 13 PD, 12 iRBD, 16 controls	Autonomic Nervous System Regulation	Enhances vagal tone and parasympathetic activity; balances sympathetic‐parasympathetic activity	Heart rate variability ↑; sleep quality ↑; relevance to RBD‐related clinical features	[90, 91, 92]
Multimodal exercise	Tango dance, yoga, tai chi, qigong, group boxing	PD clinical populations	Systematic review: 18 RCTs, 937 participants; RCTs: *n* = 44, *n* = 22, *n* = 15, *n* = 20, *n* = 20, *n* = 70	Neurocircuit & Behavioral Integration	Enhances cortical‐striatal‐brainstem connectivity; improves balance and coordination	Gait stability ↑; mood regulation ↑; sleep‐related circuit remodeling ↑	[85, 86, 87, 88, 89, 95]

Abbreviations: 6‐OHDA, 6‐Hydroxydopamin; AMPK, AMP‐activated protein kinase; BBB, blood–brain barrier; BDNF, brain‐derived neurotrophic factor; GABA, gamma‐aminobutyric acid; iRBD, isolated REM sleep behavior disorder; MAPK/ERK, mitogen‐activated protein kinase/extracellular signal‐regulated kinase; MPTP, 1‐Methyl‐4‐phenyl‐1,2,3,6‐tetrahydropyridine; NLRP3, NOD‐like receptor family pyrin domain containing 3; PD, Parkinson's disease; PFF, α‐Synuclein pre‐formed fibrils; PINK1, PTEN‐induced putative kinase 1; PSG, polysomnography; ROS, reactive oxygen species; RSWA, REM sleep without atonia; SIRT1, silent information regulator transcript 1; SLD, sublaterodorsal nucleus; TLR4, Toll‐like receptor 4; TrkB, tropomyosin receptor kinase B; UPS, ubiquitin‐proteasome system.

### 5.1 Exercise Remodeling Neurocircuit Functions

Exercise promotes neuroplasticity and may modulate neural circuits involved in sleep–wake regulation through coordinated effects across distributed brain networks. For the SLD brain region, exercise enhances GABAergic neuronal activity, helping restore balance between excitatory and inhibitory signals during the sleep–wake cycle [22]. In addition, voluntary running exercise increases the expression of glutamic acid decarboxylase 65/67 and potassium chloride co‐transporter 2 to synthesize the key enzymes in GABAergic neurons and ameliorate motoneuronal hyperexcitability [111].

In vivo studies show that voluntary running exercise repairs GABAergic neuron degeneration in the SCN, normalizes core clock gene oscillations, and corrects phase‐delayed sleep‐melatonin rhythms [112]. Exercise is also proposed to reduce the hyperexcitability of LC neurons, and long‐term voluntary wheel running exercise has been shown to increase the expression of galanin in LC noradrenergic neurons. This mechanism may regulate the sleep–wake cycle and improve sleep stability in RBD [113].

At the level of circadian rhythms, regular exercise readjusts the expression of core clock genes in peripheral tissues to synchronize the central circadian rhythm and metabolic processes, without altering the output of the SCN [114]. This exercise‐induced peripheral circadian rhythm adjustment may help stabilize the sleep–wake structure, potentially reducing the instability of REM sleep that leads to RBD symptoms. Overall, exercise may enhance the functional connectivity between the cortex, striatum, brainstem, and hypothalamus, which is relevant to sleep regulation and broader network stability [99].

### 5.2 The Mechanism of α‐Syn Clearance by Exercise

As a prodromal stage of α‐synucleinopathy, iRBD is fundamentally linked to the accumulation of misfolded α‐synuclein [100]. Exercise enhances the clearance of pathological α‐syn through multiple complementary mechanisms. First of all, exercise may activate the autophagy‐lysosomal pathway by upregulating AMP‐activated protein kinase‐mediated autophagy and PTEN‐induced putative kinase 1‐dependent mitophagy, which is a key factor in promoting the clearance of α‐syn aggregates [101]. Secondly, exercise reduces intercellular transmission of α‐syn by regulating exosome‐mediated proliferation. Microglia and their exosomes, in combination with proinflammatory factors, can increase α‐syn aggregation in neurons. Exercise may change this process by altering microglial activation and reducing inflammatory factors [102, 103]. Furthermore, activating the ubiquitin‐proteasome system (UPS) function can decrease the phosphorylated α‐syn at serine‐129 level, boost proteasome activity/E3 ligases, and facilitate misfolded pS129 α‐synclearance [104, 105]. Together, these exercise‐induced mechanisms may constitute a multi‐faceted defense against α‐syn pathology in iRBD. A recent systematic review of PD animal models demonstrates that exercise training can reduce α‐syn aggregation via inhibiting the TLR/MyD88/NF‐κB pathway and NLRP3 inflammasome activation [106].

### 5.3 Exercise Regulates Oxidative Stress and Mitochondrial Function

Regular exercise has been reported to enhance mitochondrial biogenesis, improve mitochondrial dynamics, and reduce oxidative stress [107]. Treadmill training exercise activates the nuclear factor erythroid 2 related factor 2 antioxidant pathway, upregulates heme oxygenase‐1 and γ‐glutamylcysteine ligase, limiting 1‐methyl‐4‐phenylpyridinium‐induced oxidative damage to the nigrostriatal system [108]. In the 6‐OHDA‐induced rat model, regular treadmill running exercise reduces striatal nitrite and lipid peroxidation while improving motor function [109]. Long‐term running exercise enhances mitochondrial DNA repair function by increasing mitochondrial 8‐oxoguanine DNA glycosylase‐1 levels and reducing 8‐oxo‐guanine under oxidative load [115]. Treadmill training improves redox metabolism and electron transfer in the MPTP mouse model by upregulating mitochondrial import proteins expression, thereby decreasing α‐syn aggregation, which is associated with RBD [116]. In addition, exercise normalizes mitochondrial dynamics by increasing the levels of fusion proteins optic atrophy protein 1 and mitofusin 2 in the PD mouse model [117]. These findings suggest that exercise may help repair mitochondrial damage to slow neurodegeneration and potentially improve RBD. Meta‐analyses of exercise interventions in PD have demonstrated significant improvements in sleep quality and physical function, based on pooled data from 3274 participants across Randomized Controlled Trials (RCTs) [110]. These large‐scale quantitative syntheses provide good evidence that exercise benefits sleep‐related outcomes in PD, although direct evidence in iRBD remains to be proven.

### 5.4 Exercise Reduces Neuroinflammation and Increases BDNF Modulation

Exercise may modulate RBD‐related pathology by concurrently enhancing neurotrophic support and suppressing neuroinflammation. BDNF, whose expression in the prefrontal cortex is closely linked to REM sleep integrity, is elevated by exercise. This increase activates the tropomyosin receptor kinase B and its downstream Mitogen‐Activated Protein Kinase/Extracellular Signal‐Regulated Kinase (MAPK/ERK) pathway, which is critical for stabilizing sleep architecture, including slow‐wave and REM sleep [118]. This microglial polarization is associated with the decreased levels of pathological α‐synuclein, a key protein implicated in RBD. A systematic review of animal studies in PD further confirmed that exercise training reduces pro‐inflammatory cytokines IL‐1β and TNF‐α, while increasing anti‐inflammatory IL‐10 and TGF‐β within the nigrostriatum [106]. Furthermore, exercise suppresses the activation of the NOD‐, LRR‐, and pyrin domain‐containing protein 3 inflammasome via the Toll‐like receptor 4/Myeloid differentiation factor 88/Nuclear factor kappa‐light‐chain‐enhancer of activated B cells pathway, thereby alleviating neuroinflammation‐induced dysfunction of dopaminergic neurons [58]. Collectively, by rebalancing the neuroimmune microenvironment and enhancing BDNF signaling, exercise may mitigate the inflammatory processes and synaptic instability that underpin RBD progression.

## 6 Acknowledging Limitations and Future Directions

There is very little information regarding the therapeutic advances for RBD so far. Most data are derived from PD cohorts, where sleep outcomes are secondary, and randomized controlled trials in iRBD are rare. It is believed that the optimal exercise may help RBD, but the detailed parameters of exercise such as modality, intensity, and duration remain undefined. Future studies should prioritize well‐designed trials in iRBD cohorts, incorporating objective endpoints of RSWA. The integration of multi‐omics approaches is critical for identifying exercise‐responsive biomarkers and neuroprotection. Ultimately, harnessing exercise as a strategy could fundamentally improve neurorehabilitation paradigms for patients at this critical prodromal stage of α‐synucleinopathy.

## 7 Conclusion

In conclusion, this review synthesizes current evidence suggesting that exercise represents a safe, cost‐effective, and multi‐targeted intervention with potential relevance to RBD‐related conditions. We have outlined how distinct exercise improvements may confer benefits through complementary mechanisms: exercise regulates sleep architecture, brainstem neural circuits and circadian rhythm, enhances α‐syn clearance, mitochondrial function, and reduces neuroinflammation. Based on a few studies that directly evaluate exercise as a neuroprotective effect in patients with iRBD, and more studies from PD with RBD, we propose that exercise might be a supportive intervention for RBD.

## Author Contributions

W.L. and Y.H. conceived the idea and designed the study. X.Q., Y.H. and Z.W. wrote the main manuscript text. W.L., Y.H. and R.X. critically revised the manuscript. All authors reviewed and approved the final version.

## Funding

This work was supported by the National Natural Science Foundation of China (82501716, 32220103006) and the National Key Science and Technology Project‐Changping National Laboratory Research Project (2025B‐07‐10).

## Conflicts of Interest

The authors declare no conflicts of interest.

## Data Availability

Data sharing not applicable to this article as no datasets were generated or analysed during the current study.
